# Mobile Health Apps for Improvement of Tuberculosis Treatment: Descriptive Review

**DOI:** 10.2196/17246

**Published:** 2020-04-21

**Authors:** Lina Keutzer, Sebastian G Wicha, Ulrika SH Simonsson

**Affiliations:** 1 Department of Pharmaceutical Biosciences Uppsala University Uppsala Sweden; 2 Department of Clinical Pharmacy Institute of Pharmacy University of Hamburg Hamburg Germany

**Keywords:** mHealth, video observed treatment, eHealth, model-informed precision dosing, MIPD, tuberculosis, mobile apps, therapeutic drug monitoring, smartphone

## Abstract

**Background:**

Mobile health (mHealth) is a rapidly emerging market, which has been implemented in a variety of different disease areas. Tuberculosis remains one of the most common causes of death from an infectious disease worldwide, and mHealth apps offer an important contribution to the improvement of tuberculosis treatment. In particular, apps facilitating dose individualization, adherence monitoring, or provision of information and education about the disease can be powerful tools to prevent the development of drug-resistant tuberculosis or disease relapse.

**Objective:**

The aim of this review was to identify, describe, and categorize mobile and Web-based apps related to tuberculosis that are currently available.

**Methods:**

PubMed, Google Play Store, Apple Store, Amazon, and Google were searched between February and July 2019 using a combination of 20 keywords. Apps were included in the analysis if they focused on tuberculosis, and were excluded if they were related to other disease areas or if they were games unrelated to tuberculosis. All apps matching the inclusion criteria were classified into the following five categories: adherence monitoring, individualized dosing, eLearning/information, diagnosis, and others. The included apps were then summarized and described based on publicly available information using 12 characteristics.

**Results:**

Fifty-five mHealth apps met the inclusion criteria and were included in this analysis. Of the 55 apps, 8 (15%) were intended to monitor patients’ adherence, 6 (11%) were designed for dosage adjustment, 29 (53%) were designed for eLearning/information, 3 (6%) were focused on tuberculosis diagnosis, and 9 (16%) were related to other purposes.

**Conclusions:**

The number of mHealth apps related to tuberculosis has increased during the past 3 years. Although some of the discovered apps seem promising, many were found to contain errors or provided harmful or wrong information. Moreover, the majority of mHealth apps currently on the market are focused on making information about tuberculosis available (29/55, 53%). Thus, this review highlights a need for new, high-quality mHealth apps supporting tuberculosis treatment, especially those supporting individualized optimized treatment through model-informed precision dosing and video observed treatment.

## Introduction

Tuberculosis is an infectious disease caused by *Mycobacterium tuberculosis,* which usually affects the lungs. In 2018, the World Health Organization (WHO) reported a global tuberculosis incidence of 10 million [1]. With approximately 1.5 million fatalities each year [1], tuberculosis remains one of the most common causes of death from an infectious disease worldwide. The treatment success rate for new and recurrent tuberculosis cases was estimated at 85% globally in 2017 [1]. This means that a substantial number of patients still fail to respond to treatment, have a relapse of disease, or develop drug-resistant tuberculosis [1]. There are multiple reasons for an unsuccessful treatment outcome, such as suboptimal plasma concentrations of tuberculosis drugs [2], lack of patient adherence [3], difficulties in diagnosis, or lack of education [4]. Mobile health (mHealth) apps could be valuable tools to overcome these challenges in tuberculosis treatment. Despite accumulating evidence that improving patient adherence through mobile technologies has a positive impact on treatment outcome [5-9], a clinical benefit remains to be proven for mobile interventions with respect to individualized dosing, patient education, and diagnosis.

This aim of this review was to discover, describe, and categorize Web-based and mHealth apps related to tuberculosis on the market. In 2016, Iribarren et al [10] published a review and evaluation on this matter; however, given the rapid development and further expansion of mHealth [11], an update on the topic is needed.

## Methods

### Search Strategy

The PubMed database, Google Play Store, Apple Store, Amazon, and Google were searched extensively in Sweden between February and July 2019 using the keywords “TB,” “Tuberculosis,” “Tuberkulos,” “Tuberkulose,” “Tuberculose,” “TDM,” “Therapeutic Drug Monitoring,” “Model-informed precision dosing,” “Decision-support software,” “Clinical pharmacokinetics,” “Dosing,” “Individualized dosing,” “Personalized medicine,” “Dose calculator,” “VOT,” “VDOT,” “videoDOT,” “eDOT,” “video observed treatment,” and “virtually observed treatment”. The keywords were selected by searching the literature for reviews dealing with mobile interventions for tuberculosis treatment and video observed treatment (VOT). Furthermore, studies and reviews from references in previously discovered sources were included. The search was conducted according to the Preferred Reporting Items for Systematic Reviews and Meta-Analyses (PRISMA) guidelines [12].

### Inclusion and Exclusion Criteria

mHealth apps in all languages were included if they focused on active or latent tuberculosis and were excluded if they were dedicated to other infectious diseases, or if they were not created for health improvement (eg, games).

### Description of Discovered Mobile Health Apps

Discovered mHealth apps are summarized in tables in Multimedia Appendix 1, which are classified based on the following characteristics: description, intended end user, cost, available languages, downloads, store, country developed, and qualification as a medical device. For evaluation of apps intended for therapeutic drug monitoring (TDM) and model-informed precision dosing (MIPD) for available tuberculosis drugs, the provided output from the program, electronic health record (EHR) integration, and required training were additionally assessed. All identified mHealth apps were assessed based on the information provided in the app stores, the product’s webpage, or accompanying publications.

## Results

### Apps Identified and Classification

**Figure 1:**
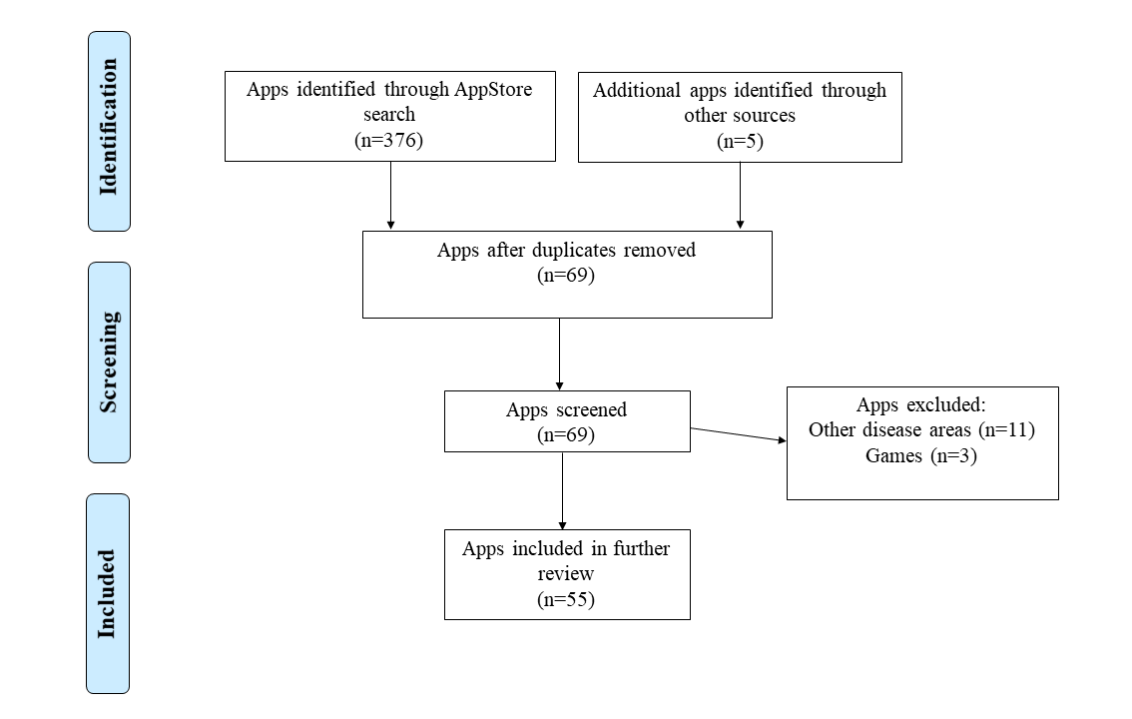
Flow chart of search strategy following Preferred Reporting Items for Systematic Reviews and Meta-Analyses (PRISMA) guidelines.

Our search identified a total of 376 mHealth apps according to the selected keywords. After removal of duplicates and irrelevant apps, 69 apps were screened and assessed in detail, 11 of which were excluded because they were focused on other disease areas, and 3 were excluded because they were games unrelated to tuberculosis. Finally, 55 mHealth apps were included in this review (Figure 1).

The 55 mHealth apps meeting the inclusion criteria were categorized (Table 1) and a descriptive, qualitative analysis was conducted. A detailed summary of all apps reviewed is provided in Multimedia Appendix 1 categorized according to intended use: monitor patients’ adherence, dosage adjustment, eLearning/information on tuberculosis, tuberculosis diagnosis, and other purposes. Each category is described in detail below.

**Table 1:** Categorization of the included mHealth apps (N=55).

Category	Description	n (%)	Examples
Adherence monitoring	Assistance for patients to keep track of their daily drug intake; video observed treatment facilitates directly observed treatment	8 (15)	miDOT – EMOCHA; AiCure; SureAdhere; TBmCure; Wisepill evriMED
Individualized dosing	Assistance for doctors with dosage adjustments based on certain patient characteristics; dosage calculators based on guidelines	6 (11)	DoseMeRx; MwPharm; InsightRx; TDMx
eLearning/information	Depiction of official guidelines; information about the disease itself and its therapy; assistance for clinicians and students to learn about tuberculosis diagnosis and disease management; display of news in the field of tuberculosis	29 (53)	Tuberculosis TB treatment and Plan; ExplainTB; Tuberculosis TB Symptoms, Causes & Diet Help; TB mobile
(Self-) Diagnosis	Diagnostics based on data input (eg, questionnaire answers, cough sound); apps for clinicians and students to train in tuberculosis diagnosis	3 (6)	TuberSpot; TimBre for Tuberculosis (TB); Diagnosa Tuberkulosis (TB)
Others	Evaluation of treatment costs; tracing of people having contact with an infected person; monitoring and tracking of patients with tuberculosis; assistance with data gathering; creation of laboratory reports	9 (16)	CAD4TB; eDetection; TB eHealth; EdetectTB; LTBI Care

### Adherence Monitoring

VOT (synonyms: video directly observed treatment, electronically directly observed treatment, video directly observed treatment, mobile directly observed treatment) describes the daily remote observation of drug intake using a smartphone app. There are two strategies to perform VOT: synchronous and asynchronous VOT. Synchronous VOT involves a live video call between patients and health care personnel. In asynchronous VOT, patients record a video of themselves, which is then stored and forwarded for later viewing by the provider. Advantages of asynchronous VOT compared to synchronous VOT are that it can be used outside of business hours and no time with the provider has to be scheduled [6,7].

Out of the 8 mHealth apps for adherence control, 3 are intended for VOT (miDOT-EMOCHA, SureAdhere, and AiCure).

MiDOT is a medication adherence app developed by emocha Mobile Health Inc (Baltimore, MD, USA). The app uses asynchronous VOT technology (store-and-forward), and care teams then review the videos and engage daily. Patients can report side effects, and the app has an additional function to filter patients struggling with adherence or experiencing side effects. The app can be purchased for Android and Apple [13].

SureAdhere is an mHealth app for asynchronous VOT. The app development was initiated by Richard Garfein from the University of California San Diego, USA. The app is compatible with Apple and Android and must be purchased. Features include patient text message or email reminders, notification to providers after a missing dose, side effect reporting, and report generation [14].

AiCure is an mHealth app using artificial intelligence to confirm medication ingestion. The software captures video, audio, and behavioral data. This app has been used in clinical trials and for population health to ensure patient adherence. The built-in algorithms have been validated against plasma blood levels. The software gathers data that can then be reviewed by health care staff [15]. In contrast to the other two main VOT tools on the market (miDOT, SureAdhere), the software of AiCure itself evaluates correct drug intake, which means that no human review is needed. Therefore, the technology is designated as automated directly observed treatment rather than VOT. The app identifies facial ID, the medication, and its ingestion. Improper administration or missed doses trigger alerts to health care workers [16]. Features of the app include reminders, interactive assistance, real-time communication with clinicians, EHR integration, and display of treatment progress [15].

Wisepill evriMED is a VOT-like app, which is connected to a smart pill dispenser that registers opening of the pillbox and subsequently sends a signal to the app. This adherence monitoring app is classified as an electronically directly observed treatment solution [17].

Other apps developed to facilitate patients’ daily drug intake include Adhere2Tx-TB, Stop TB, TBmCure, and Sembuh TB, which alert and remind patients to take their medication on a regular basis (see Multimedia Appendix 1).

### Individualized Dosing

The 6 mHealth apps providing health care professionals with assistance in dose optimization are TB Doctor, Medical Management of MDR-TB, DoseMeRx, MwPharm, InsightRx, and TDMx.

TB Doctor and Medical Management of MDR-TB are mHealth apps that enable clinicians to calculate individual doses depending on a patient’s body weight with the help of dosing tables based on current guidelines both for drug-susceptible and resistant TB.

The remaining four tools (DoseMeRx, MwPharm, InsightRx, TDMx) have been developed for dose individualization of TB medication at the bedside based on more information than body weight (Table 2).

DoseMeRx is a decision-support software for precision dosing using MIPD. The commercial cloud-based Web app, which is also available as a mobile app for Android and Apple, uses several published clinically validated population pharmacokinetic models for the calculation of individualized doses to reach the therapeutic target. All patient data are stored in a patient file and EHR integration is also possible. The software is registered as a class l medical device in Europe and Australia [18]. DoseMeRx is currently used in public and private hospitals, as well as in many teaching institutions worldwide [19], and is considered to be very user-friendly [20].

**Table 2:** Overview of mHealth apps for model-informed precision dosing of tuberculosis drugs.

Feature	DoseMeRx	MwPharm	InsightRx	TDMx
Available tuberculosis drugs	linezolid, bedaquiline, isoniazid, rifampicin, pyrazinamide, ethambutol, para-aminosalicylic acid, moxifloxacin, levofloxacin	isoniazid, rifampicin, ethambutol, streptomycin	ciprofloxacin, linezolid, rifampicin, meropenem, amikacin	meropenem, amikacin, rifampicin
Compatibility	Mac, Windows, Linux, Android, iOS	Cloud-based platform	Cloud-based platform	Cloud-based platform
Output from program	Doses and pharmacokinetic parameter estimates	Doses and pharmacokinetic parameter estimates	Doses and pharmacokinetic parameter estimates	Doses and pharmacokinetic parameter estimates
Electronic health record integration	Yes: EPIC App Orchard, Cerner Millennium, Allscripts	Yes	Yes: EPIC App Orchard, Cerner Millennium, Meditech, Centricity	No
Availability	Web-based	Web-based	Web-based	Web-based
Required training	Minimal	Minimal	Minimal	Minimal
Further information (reference)	[18]	[22]	[24]	[29]
Cost	Available at [18]	1250 Euro per license	Not publicly available	Free
Medical device	Yes	Yes	No	No

MwPharm (Mediware a.s.) was developed in 1982 at the University of Groningen, the Netherlands [21]. The Web-based version can be used from any device, including smartphones. MwPharm is a decision-support software and is therefore classified as a class l medical device [22]. The clinical value of MwPharm has been proven at the majority of Dutch hospitals and has been declared a Dutch standard by the Dutch Association of Hospital Pharmacists. Furthermore, a review from 2013 by Fuchs et al [23] comparing TDM software ranked MwPharm in the leading position. Pharmacokinetic data are analyzed using either a Bayesian approach or nonlinear regression. It is possible to modify the implemented models or to add a new model [23]. Features of the software include storage of patient records, EHR integration, report generation, genotype/phenotype analysis, and availability of models for different patient groups [22].

InsightRx (San Francisco, CA, USA) is a cloud-based Web app for precision dosing using a Bayesian approach; all implemented models are clinically validated [24]. InsightRx is considered to be very comprehensive, easy to use, and visually rich [20]. The main features include patient file storage, tracking of dosing practices across institutions, dosage history, dosing reference tables, printable reports, and constant model improvement using data from institutions [24,25].

TDMx is a cloud-based platform for precision dosing using a Bayesian approach (lead developer: Sebastian Wicha, University of Hamburg, Germany) [26]. The software is Web-based and can be freely accessed from any device with an internet connection. No data are stored in the cloud platform. Dosing can be optimized in several ways. Using the “Probabilistic Dosing” module, a likely effective first dose can be calculated using Monte Carlo simulations. When TDM data are available, the “Bayesian Dosing” module can be used to derive precision dosing based on Bayesian forecasting using TDM data. The “Optimal Sampling” module can be used as a guide to optimal sampling time points. The population pharmacokinetic models implemented in TDMx are successively validated against clinical data (eg, [27,28]).

### eLearning and Information

A total of 29 mHealth apps were identified that focus on providing patients and health care professionals with information on tuberculosis. Most of these apps provide information related to causes, risk factors, symptoms, diagnostics, treatment, or diet. The apps mainly depict treatment guidelines, provide doctors and students the opportunity to improve their skills in tuberculosis treatment, or explain the disease and its therapy to patients (see Multimedia Appendix 1). Problems were detected in many of these apps, including spelling or grammar mistakes (eg, Tuberculosis Disease [popularp, Nigeria]), wrong information (eg, in Tuberculosis [TB] [Rikki], under “description of Tuberculosis,” diabetes mellitus is described), did not include up to date information based on current guidelines or presented potentially harmful information (eg, in Tuberculosis TB Home Remedies [StatesApps, USA], it is stated that “custard apple can help to cure tuberculosis to a large extent […] and rejuvenate the drugs that are delivered for curing tuberculosis”).

### Diagnostics

Three mHealth apps (TuberSpot, TimBre for Tuberculosis [TB], Diagnosa Tuberkulosis [TB]) supporting health care professionals and patients with a TB diagnosis were included in this review.

TuberSpot (SpotLab, Spain) is a game to identify tuberculosis bacilli in samples. This app teaches the user about shape, color, clusters, and how to differentiate tuberculosis bacilli from artifacts [10]. TimBre for Tuberculosis (TB) (Rahul Pathri, India) is a screening tool in which patients are asked to cough into their smartphone microphone and are then referred to a physician if necessary. The Indonesian app Diagnosa Tuberkulosis (TB) (Informatika Unsada, Indonesia) includes features to determine the likelihood of infection.

### Other Apps

Nine mHealth apps could not be categorized within the above-mentioned categories. Their functionalities ranged from simulation of treatment costs (CAD4TB [Interactive Health Solutions, Pakistan]) [10], tracing of people that have been in contact with infected patients (eDetection [Operation Asha, India]) [10], tracking and monitoring of patients with tuberculosis (eCompliance [Operation Asha, India]), enabling researchers to identify chemical structures with activity against *M. tuberculosis* (TB mobile [Collaborative Drug, USA]) [30], facilitating data gathering by health care personnel (EdetectTB [CTMobi srls, Italy], Smart TB Puskesmas Andalas Padang [Puskesmas Andalas Padang, Indonesia], LTBI Care [WHO]), and the creation of laboratory reports (TB eHealth [iMoSyS, Malawi]).

## Discussion

### Principal Findings

Fifty-five mHealth apps related to tuberculosis were included in this review. In comparison to previous work on the topic [10], the amount of mHealth apps in the field of tuberculosis has more than doubled since 2016, which demonstrates how fast mHealth is advancing. Clearly, these findings are affected by differing inclusion criteria, but since they were comparable to the review by Iribarren et al [10], it is still reasonable to hypothesize that the amount of apps on the market has increased substantially over the past 3 years.

### Adherence Monitoring

Adherence is a major challenge in tuberculosis therapy since the treatment length ranges from 6 months for drug-susceptible tuberculosis [4] to 18 months or more in cases of drug-resistant tuberculosis [31]. Additionally, side effects can severely limit quality of life [32]. However, adherence is particularly crucial for tuberculosis treatment since interruptions in therapy can lead to suboptimal plasma drug concentrations and consequently to the development of drug-resistant bacteria and finally treatment failure [3]. According to the WHO, cases of drug-resistant tuberculosis are increasing [1], which is partly caused by a lack of patient adherence [3]. There are several approaches to improve tuberculosis patients’ adherence. One method is directly observed treatment to control drug administration. This technique has been developed further by the introduction of VOT [4].

Studies comparing directly observed treatment and VOT show that VOT is convenient for patients and providers, time and cost-effective, widely accepted among patients and health care personnel, and flexible [6,8,32]. The WHO has thus recommended the use of VOT since 2017 [4].

Potential drawbacks of VOT could be less frequent interaction between patients and providers, and therefore a lack of side effect detection, that some patients might not have access to internet or smartphones, and that it requires a secure data and video transfer [33]. Three smartphone apps are currently available for VOT (miDOT-EMOCHA, SureAdhere, AiCure).

### Individualized Dosing

A substantial number of patients still fail to respond to treatment, have a relapse of disease, or develop drug-resistant tuberculosis [1]. One of the multiple reasons for an unsuccessful treatment outcome are suboptimal plasma concentrations of tuberculosis drugs [2]. One strategy to avoid insufficient plasma concentrations, and therefore enhance cure rates, is MIPD, which is an approach to obtain individual pharmacokinetic parameters using both a priori data from population pharmacokinetic models and individually measured drug concentrations to adjust future treatment. To calculate an initial dose, patient covariates (eg, age, weight, genotype) are utilized [34-36]. Several mHealth apps have been developed as an aid for health care professionals with dosage adjustments of tuberculosis drugs. Out of the six tools currently on the market, four (DoseMeRx, MwPharm, InsightRX, TDMx) are designed for MIPD of tuberculosis medication. mHealth apps performing MIPD of tuberculosis drugs enable dosage calculation at bedside, which is very convenient for health care personnel, representing an important contribution to the improvement of tuberculosis treatment [20].

### eLearning and Information About Tuberculosis

The majority of the included apps focused on education for patients and health care professionals. Although patient education is certainly of great importance [4], it should be noted that many of these apps included errors such as grammar or spelling mistakes, or communicated false or potentially harmful information. mHealth apps without a designation as a medical device must therefore be used with caution.

Based on our analysis, only 2 of the 55 apps (4%) are currently designated as medical devices (DoseMeRx, MwPharm), which is important to ensure the quality of mHealth apps in the European Union. In the future, once the new Medical Device Regulation is enforced, more apps claiming to have a medical purpose will have to be marketed as medical devices. This will likely lead to an increase in the number of high-quality apps for tuberculosis treatment.

### Development of the Market

Since the last review on this matter in 2016, 31 new apps have been introduced to the market, representing a 129% increase in the total number of apps available. In 2016, there were no apps for improvement of adherence [10], whereas there are currently 8 tools featuring adherence monitoring for patients with tuberculosis, which is a substantial improvement. However, there is still room for further development. As discussed above, VOT tools are very valuable and have been shown to improve treatment outcomes in patients with tuberculosis [5-9]. However, VOT apps are labor- and cost-extensive, whereas approaches involving artificial intelligence for facial recognition and verification of drug intake involve less health care personnel and costs. If validated thoroughly, these technologies could be very valuable for improvement of patient adherence monitoring. Moreover, since 2016, 6 apps designed for dose individualization have entered the market. Tools for dose individualization should, whenever possible, include both individual covariate information as well as measured drug concentrations in order to predict the next dose with high accuracy and precision [34-36]. These MIPD approaches are superior to techniques based on merely covariate information such as body weight [34-36], and thus more tools enabling clinicians to use MIPD at bedside are needed. At present, 4 tools for MIPD are available; however, there are still tuberculosis drugs such as pretomanid that have not been covered by any MIPD app. In addition, none of the dose individualization apps takes within-subject variability into account, which has been shown to improve accuracy and precision in dose predictions [37,38] (personal communication, L Keutzer). Furthermore, the usage of “big data” for dose individualization is increasingly coming into the spotlight. As more data on patient covariates and drug concentration become available, machine-learning and artificial intelligence algorithms can be used to constantly update and improve the implemented dosing algorithms [24,25]. InsightRx includes such a feature, but more apps moving into the area of artificial intelligence are needed.

The number of apps providing patients and health care personnel with information on tuberculosis has increased by 164% (from 11 to 29 apps) since 2016 [10]. This shows that there has been a huge increase in apps providing information with which the market is saturated.

The number of apps for diagnostics or other purposes has remained rather constant in the last few years. In the review from 2016 [10], 8 apps for the screening and surveillance of patients with tuberculosis were discovered, and 8 such tools are currently available. Both currently and in 2016 [10], 3 apps aiding with tuberculosis diagnosis were on the market.

One major gap that was identified during this work was the availability of languages. Most apps are only available in English (39/55, 71%). This is problematic since the highest prevalence of tuberculosis cases includes nonEnglish-speaking countries such as China or Indonesia [1]. This points to a clear need to either add additional language availability to existing apps or develop new apps in other languages.

### Limitations

There are several limitations to this work. One drawback is that apps that are not free of charge or requiring a license were not purchased and consequently not scrutinized with respect to their functionalities. However, such apps were evaluated in this work and were described based on information retrieved from the app store or accompanying publications. Although it was not the aim of this review, the apps were not rated and ranked regarding their functionality and quality, as suggested in previous work [39], but were rather merely described in order to provide an overview of existing mHealth apps currently on the market. Furthermore, we might have missed including some available apps because the databases were only searched in English. In addition, the documentation of the number of downloads (see Multimedia Appendix 1) could be a misleading indicator of an app’s success, especially if the app cannot be used without payment of a fee. The number of downloads also does not account for how long the app has been available.

### Conclusions

Although the importance of the various mHealth apps currently on the market, such as tools for VOT, MIPD, or simplification of data management, for the improvement of tuberculosis treatment cannot be discounted, the majority (53%) of apps identified in this work were merely focused on providing information about tuberculosis, and many of these exhibited issues regarding spelling, grammar, or correctness of the information provided. Thus, this review demonstrates a need for more mHealth apps of high quality supporting tuberculosis treatment.

## Appendix

Multimedia Appendix 1 Tables of mHealth applications included in the review.

## Abbreviations

EHR: electronic health record
mHealth: mobile health
MIPD: model-informed precision dosing
PRISMA: Preferred Reporting Items for Systematic Reviews and Meta-Analyses
TDM: therapeutic drug monitoring
VOT: video observed treatment
WHO: World Health Organization
