# Supplementary material for: Mobile Health Apps for Improvement of Tuberculosis Treatment: Descriptive Review
Source: JMIR Mhealth Uhealth. 2020 Apr 21;8(4):e17246. doi: 10.2196/17246 (PMC7201317; doi:10.2196/17246)
Supplement: Multimedia Appendix 1 [file mhealth_v8i4e17246_app1.docx]

**Multimedia appendix S1: Tables of mHealth apps included in the review**

Supplement to:

Title: Mobile Health Apps for Improvement of Tuberculosis Treatment: Descriptive Review

Authors: Lina Keutzer^1^; Sebastian Georg Wicha^2^, PhD; Ulrika Sigrid Helena Simonsson^1^, PhD, MSc

Affiliations:

^1^ Department of Pharmaceutical Biosciences, Uppsala University, Uppsala, Sweden

^2^ Department of Clinical Pharmacy, Institute of Pharmacy, University of Hamburg, Hamburg, Germany

# Monitoring of patient adherence

**Table S1.** Summary of mobile apps and web-based tools intended to improve the adherence of a TB patient

| Name | Store | Country developed | Available languages | Intended end-user | Costs | Medical Device? | Downloads | Description | | URL |
| --- | --- | --- | --- | --- | --- | --- | --- | --- | --- | --- |
|  |  |  |  |  |  |  |  |  |  | |
| *Adhere2Tx-TB* | Google | Georgia | Georgian | Patients | Free | No | 100+ | Facilitates everyday medication for patients enrolled in the National Georgian TB program | <https://play.google.com/store/apps/details?id=com.leavingstone.adhere&hl=en> | |
| *TBmCure* | Google | India | English | Healthcare professionals & Patients | Free | No | 500+ | Offers health care officers to track, monitor and help patients; also alerts and reminds patients if a dose is missed, and confirms the prescribed dosage taken by the patient | <https://play.google.com/store/apps/details?id=com.eighteenpixels.tbmcure&hl=en> | |
| *Sembuh TB* | Google | Indonesia | Indonesian | Patients | Free | No | 1000+ | Reminds patients to take their medication and contains articles and educational videos about tuberculosis | <https://play.google.com/store/apps/details?id=com.sembuhapp.hybrid&hl=en> | |
| *miDOT – EMOCHA* | Google,  Apple | US | English | Healthcare professionals & Patients | Free | No | 1000+ | Intended for asynchronous VOT (Video Observed Therapy). Patients can report side effects and the app has a function to filter patients struggling with adherence or experiencing side effects. | <https://play.google.com/store/apps/details?id=com.emocha.midot.a&hl=en> | |
| *SureAdhere* | Google, Apple | US | English | Healthcare professionals & Patients | Free | No | 100+ | Intended for asynchronous VOT (Video Observed Therapy). Features include patient SMS/Email reminders, notification to providers after a missing dose, side effect reporting and report generation. | <https://play.google.com/store/apps/details?id=com.sureadhere.mobile2&hl=en> | |
| *AiCure* | Google, Apple | US | English | Healthcare professionals & Patients | Free | No | 1000+ | Intended for VOT (Video Observed Therapy). Artificial intelligence is used to confirm medication ingestion. The software captures video, audio and behavioral data. It is used in clinical trials and for population health to ensure patient adherence. | <https://play.google.com/store/apps/details?id=com.aicure.aiview.clinical.master.mobile&hl=en> | |
| *Stop TB* | Google | Myanmar | not available in English | Patients | Free | No | 100+ | Pill and sputum test notification | NA | |
| *evriMED Caregiver* | Google, Apple | South Africa | English | Healthcare professionals & Patients | Free | No | 10+ | An app connected to a smart-pill dispenser which registers the opening of the pillbox and subsequently sends a signal to the app. This adherence monitoring app is classified as an eDOT (electronically directly observed treatment) solution. | <https://play.google.com/store/apps/details?id=com.wisepill.Caregiver&hl=en> | |

# Legend: TB: tuberculosis, US: United States of America

# Individualized dosing solutions

**Table S2.** Summary of mobile apps and web-based tools offering individual dosing solutions for TB treatment

| Name | Store | Country developed | Available languages | Intended end-user | Costs | Medical Device? | Downloads | Description | URL |
| --- | --- | --- | --- | --- | --- | --- | --- | --- | --- |
|  |  |  |  |  |  |  |  |  |  |
| *TB Doctor* | Google | India | English | Healthcare professionals & Patients | Free | No | 1000+ | Calculates individual dosage based on bodyweight for drug susceptible & MDR TB, summarizes side effects, lists all anti-TB drugs available on the Indian market, states regimens for drug susceptible & MDR TB | <https://play.google.com/store/apps/details?id=appinventor.ai_rsnphone.TB_Doc&hl=en> |
| *Medical Manage-ment of MDR-TB* | Google | US | English | Healthcare professionals | Free | No | 1000+ | Information about TB drugs, dosing calculator based on bodyweight, dosing tables, management of adverse events | <https://play.google.com/store/apps/details?id=org.pih.pihguidetomdr_tb&hl=en> |
| *DoseMeRx* | Google, Apple | Australia | English | Healthcare professionals | Available at: https://doseme-rx.com/pricing | Yes | 100+ | Model-informed precision dosing support tool using Bayesian Forecasting to predict the dose that results in an optimal exposure for a patient | <https://play.google.com/store/apps/details?id=au.com.doseme.dosemewebframe&hl=en> |
| *MwPharm Online ++* | Web-based applica-tion | The Nether-lands | English | Healthcare professionals | 1250 Euro per single seat license | Yes | NA | Model-informed precision dosing support tool using Bayesian Forecasting to predict the dose that results in an optimal exposure for a patient | <http://www.mediware.cz/en/mwpharm/> |
| *InsightRx* | Web-based applica-tion | US | English | Healthcare professionals | Upon request | No | NA | Model-informed precision dosing support tool using Bayesian Forecasting to predict the dose that results in an optimal exposure for a patient | <https://www.insight-rx.com/> |
| *TDMx* | Web-based applica-tion | Germany | English | Healthcare professionals | Free | No | NA | Model-informed precision dosing support tool using Bayesian Forecasting to predict the dose that results in an optimal exposure for a patient | <http://www.tdmx.eu/> |

# Legend: TB: tuberculosis, US: United States of America, MDR-TB: multi-drug resistant tuberculosis

# eLearning / information

**Table S3.** Summary of mobile apps and web-based tools for eLearning or information about TB

| Name | Store | Country developed | Available languages | Intended end-user | Costs | Medical Device? | Downloads | Description | URL | |
| --- | --- | --- | --- | --- | --- | --- | --- | --- | --- | --- |
|  |  |  |  |  |  |  |  |  |  | |
| *TBeReview DRTB* | Google | US | English | Healthcare professionals | Free | No | 100+ | Helps Healthcare professionals to improve their skills in diagnosis & management of drug-resistant TB | <https://play.google.com/store/apps/details?id=com.gisvcs.drtb.drtb&hl=en> | |
| *TBeReview HIVTB* | Google | US | English | Healthcare professionals | Free | No | 100+ | Helps Healthcare professionals to improve their skills in diagnosis & management of TB in the setting of HIV | <https://play.google.com/store/apps/details?id=com.gisvcs.hivtb.hivtb&hl=en> | |
| *TB eLearning* | Google | Bangla-desh | English | Patients | Free | No | 1+ | Education and information regarding TB | <https://play.google.com/store/apps/details?id=mpower.org.elearning_module&hl=en> | |
| *Anti-TB Drugs* | Google | NA | English | Medical students | Free | No | 500+ | List of 1^st^ & 2^nd^ line TB drugs | <https://play.google.com/store/apps/details?id=appinventor.ai_rsnphone.AntiTB2&hl=en> | |
| *Tuberculose DZ* | Google | Algeria | French | Medical students & Healthcare professionals | Free | No | 1000+ | Ebook of “The TB Handbook 2011” | <https://play.google.com/store/apps/details?id=dev.toudert.TuberculoseDz&hl=en> | |
| *Tuberculosis TB treatment and Plan* | Google | NA | English | Patients | Free | No | 1000+ | Information about TB regarding prevention, symptoms, dietary advice during active disease, diagnosis and treatment | <https://play.google.com/store/apps/details?id=com.pks_easy.Diet_Plan_for_Tuberculosis&hl=en> | |
| *ExplainTB* | Google, Apple | Germany | 36 languages | Patients | Free | No | 500+ | Provides medical information about tuberculosis and its treatment, an audio version is available | <https://www.explaintb.org/download/?lang=en> | |
| *Tuberculosis info* | Google | NA | English | Patients | Free | No | 100+ | Information about TB regarding cause, symptoms, transmission, diagnosis, prevention & vaccines, epidemiology | <https://play.google.com/store/apps/details?id=com.programmingisfun.tuberculosis&hl=en> | |
| *Tuberculosis Help-Offline* | Google | NA | English | Patients | Free | No | 1000+ | Information on causes, symptoms, risk factors, dietary advice and general prevention tips to avoid infection | <https://play.google.com/store/apps/details?id=com.aegroup.health.tuberculosis&hl=en> | |
| *Tuberkulos* | Google | Sweden | Swedish | Patients | Free | No | 100+ | Information about symptoms, causes, diagnostic etc. | NA | |
| *SNTC* | Apple | US | English | Healthcare professionals | Free | No | NA | Links to news, products, webinars and training related to TB. Developed by University of Florida. | <https://apps.apple.com/us/app/sntc/id557358242> | |
| *Tuber-culosis: Causes, Diagnosis, and Manage-ment* | Google | NA | English | Patients | Free | No | 500+ | Assists to understand the Tuberculosis Guide and describes disease management, diagnosis and causes | <https://play.google.com/store/apps/details?id=com.healthinfo.tuberculosis.cause.prevention.management.treatment.diagnosis&hl=fil> | |
| *Tuberculosis TB Symptoms, Causes & Diet Help* | Google | NA | English | Patients | 25-55 SEK | No | 5000+ | Information on causes, risk factors, types of tuberculosis, symptoms in infants and adults, transmission and dietary advice for TB patients | <https://play.google.com/store/apps/details?id=com.OneLife2Care.TuberculosisTBHelp&hl=fil> | |
| *TB Clinical guide* | Apple, Google | South Africa | English | Healthcare professionals | Free | No | 10000+ | Presents National TB guidelines (South Africa) for healthcare professionals (National Tuberculosis Management Guidelines 2014, MDR-TB Clinical Guidelines (Updated January 2013) and National Childhood TB Guidelines 2013) | | <https://play.google.com/store/apps/details?id=omp.guidance.tb&hl=fil> |
| *Nursing Care for TB* | Google | Indonesia | English | Patients | Free | No | 100+ | Information about TB | <https://m.apkpure.com/group/com.nursing.caretb?posts=1> | |
| *How to prevent Tuberculosis* | Google | NA | English | Patients | Free | No | 100+ | Provides advice on how to prevent TB, BCG vaccine and healthy lifestyle | <https://play.google.com/store/apps/details?id=com.andromo.dev717872.app788049&hl=en> | |
| *Tuberculosis: Causes, Symptoms Signs & Diagnosis* | Google | NA | English | Patients | Free | No | 10+ | Information about TB causes, symptoms & signs, prevention, *M. tuberculosis*, diet management, treatment and diagnosis | <https://play.google.com/store/apps/details?id=com.thereyv.tuberculosis&hl=en> | |
| *Tuberculosis Disease* | Google | NA | English | Patients | Free | No | 100+ | Information about TB regarding causes, symptoms, cure, treatment and diagnosis | <https://play.google.com/store/apps/details?id=com.andromo.dev542547.app880354&hl=en> | |
| *Tuberculosis* | Google | NA | English | Patients | Free | No | 500+ | Information about TB | <https://play.google.com/store/apps/details?id=com.andromo.dev589470.app574580&hl=en> | |
| *N-TB* | Google | India | English | Healthcare professionals | Free | No | 1000+ | Enables healthcare workers to assess the nutritional status of TB patients, counsel tips on diet and daily calorie intake,  estimate BMI, classify the severity of undernutrition, suggests clinical actions based on the BMI and suggests daily caloric and protein intake for underweight patients with active TB. | <https://www.sciencedirect.com/science/article/pii/S0019570718303160> | |
| *TB mukt Himachal* | Google | India | Hindi / English | Patients & Healthcare professionals | Free | No | 100+ | Initiative by Indian government; individuals can assess risk factors and access details of all TB test facilities, information about TB | <https://play.google.com/store/apps/details?id=com.app.tbmukt&hl=en> | |
| *Tuberculosis sintomas causas y tratamiento* | Google | Italy | Spanish | Patients | Free | No | 5+ | Provides information about TB regarding causes, symptoms, prevention, types, diagnosis and treatment | <https://play.google.com/store/apps/details?id=com.itatvadev.tuberculosissintomascausasytratamiento&hl=en> | |
| *OneImpact* | Google, Apple | NA | English, Dutch, French, German, Italian, Portu-guese | Patients | Free | No | 100+ | Provides patients with information about TB and gives them access to medical services. A chat function to report problems is also available. | <https://play.google.com/store/apps/details?id=com.duretechnologies.android.ios.android.maxico&hl=en> | |
| *Tuberculosis TB Home Remedies* | Google | NA | English | Patients | Free | No | 1000+ | Presents home remedies against TB, mainly focused on custard apple | <https://play.google.com/store/apps/details?id=com.StatesApps.RemedyTuberculosis&hl=en> | |
| *Tuberculosis Symptoms Guide* | Ama-zon | NA | English | Patients | Free | No | NA | A symptom guide with questionnaires for patients | <https://www.amazon.com/TEJAS-YB-TUBERCULOSIS-SYMPTOMS-GUIDE/dp/B00IMUVZKE> | |
| *Tuberculosis (TB)* | Ama-zon | NA | English | Patients | Free | No | NA | Provides information on TB | <https://www.amazon.com/Rikki-Tuberculosis-TB/dp/B017LU5MP4> | |
| *Tuberculosis* | Ama-zon | NA | English | Patients | Free | No | NA | Information on symptoms, causes, home remedies, medication, prevention, detection etc. | NA | |
| *Tuberculosis News* | Ama-zon | NA | English | Patients | $0.99 | No | NA | News about TB | NA | |
| *Tuberculosis Awareness* | Ama-zon | NA | English | NA | Free | No | NA | NA | <https://www.amazon.com/Ajay-Tuberculosis-Awareness/dp/B00VKPIM18> | |

# Legend: TB: tuberculosis, US: United States of America, MDR-TB: multi-drug resistant tuberculosis, eLearning: electronic learning

# Diagnostics

**Table S4.** Summary of mobile apps and web-based tools assisting with TB diagnosis

| Name | Store | Country developed | Available languages | Intended end-user | Costs | Medical Device? | Downloads | Description | URL |
| --- | --- | --- | --- | --- | --- | --- | --- | --- | --- |
|  |  |  |  |  |  |  |  |  |  |
| *TuberSpot* | Google, Apple | Spain | English, Spanish | Healthcare professionals | Free | No | 5000+ | Game to practice identification of *Mycobacterium tuberculosis* in samples, helps healthcare professionals to improve their diagnostic skills; images from real patients are used in the app | <https://play.google.com/store/apps/details?id=com.spotlab.TuberSpot&hl=en> |
| *TimBre for Tuberculosis (TB)* | Google | India | English | Patients | Free | No | 500+ | A screening tool: patients cough into their phone microphone and get results via SMS (they are then referred to a Physician for diagnostic tests) | <https://play.google.com/store/apps/details?id=com.docturnal.tbscreening&hl=en> |
| *Diagnosa Tuberkulosis (TB)* | Google | Indonesia | Indonesian | Patients & Healthcare professionals | Free | No | 100+ | Feature to determine the likelihood of infection, maps displaying hospitals, provides information about TB | <https://play.google.com/store/apps/details?id=com.ratih.skripsi_tb&hl=en> |

# Legend: TB: tuberculosis

# Others

**Table S5.** Summary of other mobile apps and web-based tools for TB treatment currently on the market

| Name | Store | Country developed | Available languages | Intended end-user | Costs | Medical Device? | Downloads | Description | URL |
| --- | --- | --- | --- | --- | --- | --- | --- | --- | --- |
|  |  |  |  |  |  |  |  |  |  |
| *CAD4TB* | Apple | Spain | English, French, Spanish | Healthcare professionals | Free | No | NA | Performs economic simulations for TB diagnostics comparing the costs of dCXR/CAD screening to Xpert® MTB/RIF | <https://apps.apple.com/us/app/cad4tb/id973833058> |
| *eDetection* | Google | India | English | Healthcare professionals | Free | No | 100+ | Assists with identification and diagnosis of people, who may have come in contact with a TB patient using contact tracing | <https://play.google.com/store/apps/details?id=org.opasha.ecompliance.econtact.tracing&hl=en> |
| *eCompliance* | Google | India | English | Healthcare professionals | Free | No | 100+ | Identification system to track and monitor TB patients | <https://play.google.com/store/apps/details?id=org.opasha.eCompliance.ecompliance&hl=en> |
| *EdetectTB* | Google | Italy | English | Healthcare professionals | Free | No | 10+ | Simplifies data gathering by healthcare personnel, data can be downloaded to a clinical database | <https://play.google.com/store/apps/details?id=com.ctmobi.edt&hl=en> |
| *TB eHealth* | Google | Malawi | Nyanja | Healthcare professionals | Free | No | 100+ | App to capture TB presumptive details and creation of laboratory results for healthcare professionals | <https://play.google.com/store/apps/details?id=com.imosys.ntcp&hl=en> |
| *EasyTB* | Google | Indonesia | Indonesian | Healthcare professionals | Free | No | 100+ | For access of PNPK (Indonesian National Guidelines for Medical Services) for healthcare professionals | <https://play.google.com/store/apps/details?id=com.gmail.easytb.easytbindonesia&hl=en> |
| *Smart TB Puskesmas Andalas Padang* | Google | Indonesia | Indonesian | Healthcare professionals | Free | No | 100+ | For reporting suspected TB cases and integration of data into a health centre database | <https://play.google.com/store/apps/details?id=com.lucgu.tb&hl=en> |
| *LTBI Care* | Google, Apple | Switzer-land | English | Healthcare professionals | Free | No | 100+ | The World Health Organization with support from the European Respiratory Society has developed an app for management of latent tuberculosis. The app contains features to collect patient information and to monitor and manage at-risk populations. | <https://play.google.com/store/apps/details?id=com.duretechnologies.apps.android.ltbi_care&hl=en> |
| *TB mobile* | Google | US | English | Healthcare professionals &  Patients | Free | No | 1000+ | Database of chemical structures exhibiting activity against *Mycobacterium tuberculosis* on known targets, including the feature to search for molecules which potentially infer with targets | <https://play.google.com/store/apps/details?id=com.tbeye.android.tbmobile&hl=en> |

# Legend: TB: tuberculosis, US: United States of America
